# Risk factor analysis and predictive model development for healthcare-associated infections post-coronary artery bypass grafting

**DOI:** 10.3389/fpubh.2025.1605272

**Published:** 2025-07-03

**Authors:** Yan Liu, Lingbo Xue, Ping Jiang, Jiaojiao Shen, Xiao Peng, Xiaoqiang Yu

**Affiliations:** ^1^Infection Management Department, Nantong First People's Hospital, Nantong, China; ^2^Department of Cardiovascular Surgery, Nantong First People's Hospital, Nantong, China

**Keywords:** coronary artery bypass grafting (CABG), health care associated infections (HAIs), logistic regression, prediction model, risk factors

## Abstract

**Objective:**

This study aimed to analyze the risk factors associated with healthcare-associated infections (HAIs) in individuals who underwent post-coronary artery bypass grafting (CABG) and to develop a predictive model for infection risk assessment.

**Methods:**

Clinical data were retrospectively collected from patients who underwent CABG at our hospital between January 2019 and December 2023. Data sources included the hospital infection surveillance system, hospital information system, and a questionnaire for HAIs in patients after cardiac surgery. Patients were divided into an infection group and a non-infection group based on whether they developed HAIs during the postoperative hospitalization period. Logistic regression was used to identify independent risk factors and to develop a risk prediction model. The predictive performance of the model was assessed using receiver operating characteristic curve analysis.

**Results:**

Independent risk factors for HAIs post-CABG included diabetes (odds ratio [OR] = 1.467), preoperative white blood cell count (OR = 0.117), preoperative albumin levels (OR = −0.146), intraoperative blood transfusion (OR = 0.001), presence of an indwelling drainage tube (OR = 0.864), drainage volume (OR = 0.003), duration of ventilator use (OR = 0.656), and central venous catheterization time (OR = 0.103). The predictive model was established as: Ln (P/1−P) = −2.230 + 1.467 * diabetes + 0.117 * preoperative white blood cell count −0.146 * preoperative albumin + 0.001 * intraoperative blood transfusion + 0.864 * drainage tube indwelling + 0.003 * drainage volume + 0.656 * ventilator use time + 0.103 * central venous catheterization time. The Hosmer-Lemeshow test indicated a good model fit with observed values. Receiver operating characteristic curve analysis demonstrated that the model achieved an area under the curve of 0.970, with a sensitivity of 90.5% and a specificity of 92.1%.

**Conclusion:**

The independent risk factors for HAIs after CABG were diabetes, body mass index, preoperative white blood cell count, intraoperative blood transfusion volume, duration of pericardial and mediastinal drainage tube placement, total drainage volume, duration of mechanical ventilation, and duration of central venous catheterization. The developed risk prediction model demonstrated high accuracy in estimating postoperative HAI risk.

## 1 Introduction

Healthcare-associated infection (HAI), also referred to as nosocomial infection, has emerged as a significant public health concern worldwide (1). China’s aging population has contributed to a rising incidence of coronary atherosclerotic heart disease (CHD) posing substantial challenges to the prevention and management of cardiovascular diseases globally (2). Coronary artery bypass grafting (CABG) is widely recognized as one of the most effective interventions for the treatment of coronary heart disease (3). In recent years, the frequency of CABG procedures has increased considerably (4). However, due to the complexity of the surgical procedure, the extended operative duration, and its invasive nature, CABG is associated with higher likelihood of postoperative complications, including HAIs (5). These complications not only adversely impact surgical outcomes and patient recovery but may also pose life-threatening risks (6). Consequently, reducing postoperative complications post-CABG has become a focal point of research and clinical practice.

Studies have indicated that individuals undergoing CABG are at an elevated risk of developing HAIs, which represents one of the most common postoperative complications, with an infection rate as high as 20% (7, 8). Postoperative infections can adversely impede recovery and surgical outcomes, potentially leading to prolonged hospitalization, increased healthcare costs, and in severe cases, mortality (9). As a result, early identification of risk factors and accurate prediction of HAI risk is critical for implementing preventive strategies and improving patient outcomes.

Research on HAIs in individuals post-CABG in China remains relatively limited. Although some studies have been conducted, findings are fragmented and comprehensive investigations on risk prediction models remain scarce. Existing studies on risk factors are not exhaustive and a well-established theoretical framework and research methodology are lacking. The logistic regression model has been widely utilized in clinical risk prediction due to its ability to effectively demonstrate the relationships between independent and dependent variables, providing valuable predictive insights into disease occurrence (10).

The present study aimed to systematically evaluate the prevalence and independent risk factors of HAIs in individuals undergoing CABG and to develop a predictive risk model. The primary objective was to facilitate the early identification of HAI risk factors, guide the implementation of targeted infection prevention strategies, and effectively reduce the incidence of HAIs post-CABG. Additionally, new perspectives and methodologies were contributed by this study to enhance postoperative infection prevention and control in patients undergoing CABG.

## 2 Study participants and methods

### 2.1 Study participants

This observational study included a total of 445 individuals who underwent CABG at the hospital between January 2019 and December 2023. This study was conducted in accordance with the declaration of Helsinki and approved by the Ethics Committee of Nantong First People’s Hospital. Written informed consent was obtained from all participants.

Inclusion criteria were as follows: ① Individuals who met the diagnostic criteria for coronary heart disease and had a confirmed diagnosis through coronary angiography prior to surgery; ② Individuals aged 18 years or older; ③ Individuals with no history of cardiac surgery; ④ Individuals who underwent off-pump CABG as the surgical approach.

Exclusion criteria were as follows: ① Individuals with a community-acquired infection at the time of admission; ② Individuals with preoperative history of valvar heart disease and congenital heart disease; ③ Individuals who had recently used antibacterial agents; ④ Individuals with incomplete clinical data.

### 2.2 Diagnosis of HAI

HAI diagnosis was determined in accordance with the criteria outlined in the Diagnostic Criteria for HAI (Trial) issued by the Ministry of Health of China (11). Additionally, to ensure accuracy, a second review was conducted by HAI specialists and medical experts in cardiac and vascular surgery to confirm the presence of HAIs during the postoperative hospital stay. Based on this evaluation, individuals were categorized into either an infected group or a non-infected group.

### 2.3 Questionnaire contents

#### 2.3.1 Socio-demographic data

The questionnaire collected socio-demographic information, including inpatient number, age, sex, ward, preoperative hospital stay (days), postoperative hospital stay (days), total hospital stay (days), past medical history, body mass index (BMI), and other relevant details.

#### 2.3.2 Preoperative biochemical indicators

Preoperative biochemical indicators were recorded, such as albumin (g/L), white blood cell count (×109/L), and other pertinent laboratory values.

#### 2.3.3 Surgery-related factors

Surgery-related factors included surgical approach, anesthesia method, duration of surgery(hours), American Society of Anesthesiologists (ASA) surgical risk classification, National Nosocomial Infections Surveillance (NNIS) surgical risk classification, surgical grade, intraoperative blood transfusion, and type of surgical incision.

#### 2.3.4 Invasive procedures

Data on invasive procedures were collected, including presence of drainage tube, tracheal intubation, and other relevant interventions.

#### 2.3.5 Medication-related factors

Medication-related factors encompassed the use of prophylactic antibiotics, intraoperative antibiotic administration, and other related pharmacological details.

#### 2.3.6 HAI status

HAI status included information such as site of infection, pathogenic bacteria, and others.

### 2.4 Methods

#### 2.4.1 Data collection

In alignment with the target monitoring requirements for HAIs, a structured questionnaire was developed, and data were collected using a real-time HAI monitoring system and the hospital information system. Case data for all enrolled individuals included demographic characteristics, medical history, left ventricular ejection fraction (LVEF), duration of surgery, extracorporeal circulation time, pathogen profiles, and other relevant clinical variables.

#### 2.4.2 Pathogen identification and antimicrobial susceptibility testing

When signs of suspected infection were observed during hospitalization post-CABG, sputum, blood, wound secretions, and samples from suspected infection sites were aseptically collected and placed in sterile sampling tubes for pathogen culture. Bacterial identification and antimicrobial susceptibility testing were performed using the BioMérieux ATB fully automated identification system, in accordance with the guidelines outlined in the National Guide to Clinical Laboratory Procedures (12). Quality control strains, including *Pseudomonas aeruginosa* ATCC28753, *Escherichia coli* ATCC25922, *Staphylococcus aureus* ATCC25923, and *Candida albicans* ATCC90028, among others, were obtained from the Clinical Laboratory Center of the Ministry of Health. Duplicate strains isolated from the same patient were excluded from the analysis.

#### 2.4.3 Quality control

Data collectors underwent prior training in HAI-related knowledge and strictly adhered to the inclusion and exclusion criteria throughout the data collection process. To further enhance data accuracy, confirmed cases were reviewed by cardiovascular surgeons and hospital infection management experts. Following data collection, entries were independently recorded and cross-checked by two individuals to ensure data authenticity.

#### 2.4.4 Statistical analysis

Statistical analysis was performed using SPSS version 23.0. Categorical variables were expressed as frequencies and percentages (%), with group comparisons conducted using the chi-square test or Fisher’s exact test. Continuous variables following a normal distribution were presented as mean ± standard deviation, and group differences were assessed using the *t*-test. Risk factors were identified using univariate and multivariate logistic regression analyses. Statistically significant variables were included in a stepwise logistic regression model to identify independent risk factors and construct a predictive model, with results expressed as odds ratios (OR) and 95% confidence intervals (95% CI). Multicollinearity among independent variables was evaluated using variance inflation factors. Model goodness-of-fit was assessed with the Hosmer-Lemeshow test, where *p* > 0.05 indicated good fit. Predictive performance was evaluated using ROC curves; the closer the area under the curve (AUC) is to 1, the better the model’s discrimination. A two-tailed test with a significance level of *α* = 0.05 was used, and *p* < 0.05 was considered statistically significant.

## 3 Results

### 3.1 Occurrence of HAI

Among the 445 patients who underwent CABG, 42 developed HAIs, resulting in an infection rate of 9.44%. A total of 46 infection cases were recorded, corresponding to an overall infection rate of 10.34%. The highest number of infections occurred in the second quarter with an infection rate of 13.83%, followed by the fourth quarter, where the infection rate was 10.88, as shown in Table 1.

**Table 1:** HAIs in patients post-CABG.

Time grouping	Number of cases monitored	Number of infected cases	Infection rate (%)	Number of times of infected cases	Infection time rate (%)
Year					
2019	103	12	11.65	14	13.59
2020	86	7	8.14	7	8.14
2021	64	6	9.38	7	10.94
2022	99	8	8.08	9	9.09
2023	93	9	9.68	9	9.68
Quarter					
1	83	6	7.22	6	7.22
2	94	11	11.70	13	13.83
3	121	10	8.26	11	9.09
4	147	15	10.20	16	10.88
Total	445	42	9.44	46	10.34

### 3.2 Distribution of HAI sites

The respiratory system was the most frequently affected HAI site among patients post-CABG, accounting for 58.70% of cases. Surgical site infections were the second most common, comprising 21.74% of cases, as detailed in Table 2.

**Table 2:** Distribution of HAI sites post-CABG.

Infection site	Number of cases (*n* = 46)	Incidence rate (%)
Respiratory system	27	58.70
Upper respiratory tract	2	4.35
Lower respiratory tract	16	34.78
Ventilator associated pneumonia	7	15.22
Pleural cavity	2	4.35
Surgical site infection	10	21.74
Superficial incision infection	5	10.87
Deep incision infection	5	10.87
Blood system	3	6.52
Septicemia	1	2.17
Catheter-associated bloodstream infection	2	4.35
Urinary system	6	13.04
Urinary tract infection	4	8.70
Catheter associated urinary tract infection	2	4.35

### 3.3 Distribution of pathogenic bacteria in HAI

Among the 46 individuals with HAIs post-CABG, a total of 56 strains of pathogenic bacteria were identified. Gram-negative bacteria were the most prevalent, comprising 38 strains (67.86%), followed by gram-positive bacteria with 12 strains (21.43%). Additionally, 6 fungal strains were detected. The detailed distribution is presented in Table 3.

**Table 3:** Species and composition ratio of pathogenic bacteria.

Pathogenic bacteria	Number of strains	Composition ratio (%)
Gram-positive bacteria	12	21.43
*Enterococcus faecium*	2	3.57
*Staphylococcus aureus*	10	17.86
Gram-negative bacteria	38	67.86
*Enterobacter aerogenes*	1	1.79
*Klebsiella pneumoniae*	14	25.00
*Escherichia coli*	6	10.71
*Pseudomonas aeruginosa*	9	16.07
*Acinetobacter baumannii*	7	12.5
*Stenotrophomonas maltophilia*	1	1.79
Fungi	6	10.71
*Candida albicans*	5	8.93
*Filamentous fungi*	1	1.79

### 3.4 Univariate analysis

Univariate analysis was conducted with HAIs as the dependent variable and associated influencing factors as independent variables. The findings demonstrated that BMI, diabetes, preoperative albumin levels, preoperative white blood cell count, preoperative C-reactive protein levels, operative duration, intraoperative blood transfusion, administration of additional antibiotics during surgery, duration of pericardial mediastinal drainage tube placement, duration of thoracic drainage tube placement, total drainage volume, total length of hospital stay, postoperative hospital stay, duration of surgical intensive care unit stay, incidence of unplanned reoperation, postoperative ventilator use duration, urinary catheterization duration, and central venous catheterization duration were significantly associated with an increased risk of HAIs post-CABG (*p <* 0.05). The detailed results are shown in Table 4.

**Table 4:** CABG univariate analysis of HAIs post-CABG.

Influencing factor	Infected group (*n* = 42)	Non-infected group (*n* = 403)	Statistic	*p value*
Age (year)	66.07 ± 7.66	63.28 ± 9.02	1.932	0.054
Sex			0.895	0.344
Male	33	289		
Female	9	114		
			3.901	0.048
	6	115		
	36	288		
Hypertension			0.772	0.380
Yes	23	192		
No	19	211		
Diabetes			5.692	0.017
Yes	26	172		
No	16	231		
Hyperlipemia			0.778	0.378
Yes	9	112		
No	33	291		
Preoperative albumin (g/L)	33.528 ± 4.755	38.031 ± 4.43	6.224	<0.001
Preoperative LVEF (%)	58.60 ± 6.38	57.0 ± 6.18	1.554	0.121
Preoperative white blood cell count (×10^9^/L)	9.82 ± 3.215	7.243 ± 4.279	−3.793	<0.001
Preoperative C reactive protein (ng/L)	23.685 ± 26.666	6.466 ± 13.263	−5.184	<0.001^a^
NNIS scoring			4.955	0.082^b^
Grade 1	0	29		
Grade 2	41	371		
Grade 3	1	3		
ASA classification			3.793	0.238^b^
II	4	18		
III	28	299		
IV	9	82		
V	1	4		
Operation duration (h)	5.168 ± 1.551	4.525 ± 1.513	−2.613	0.009
Intraoperative blood transfusion volume (ml)	549.517 ± 561.124	1413.036 ± 997.524	−6.891	<0.001^a^
Bypass number			7.567	0.094^b^
1	2	74		
2	12	120		
3	13	112		
4	12	82		
5	3	15		
Prophylactic antibiotics				0.468^b^
Yes	39	383		
No	3	20		
Addition of antibiotics during operation			4.516	0.034
Yes	1	56		
No	41	347		
Pericardial mediastinal drainage tube indwelling time (d)	5.00 ± 1.89	2.65 ± 1.09	−8.777	<0.001^a^
Thoracic drainage tube indwelling			7.583	0.006
Yes	12	52		
No	30	351		
Total drainage (ml)	1007.57 ± 239.93	672 ± 212.94	−7.474	<0.001
Aortic balloon pump				0.169^b^
Yes	5	23		
No	37	380		
Hospital stay (d)	29.17 ± 7.85	29.17 ± 10.46	−5.409	<0.001
Preoperative hospital stay (d)	9.57 ± 5.047	9.16 ± 5.096	−0.497	0.620
Postoperative hospital stay (d)	29.17 ± 10.46	22.04 ± 7.847	−4.681	<0.001^a^
sICU retention time (d)	3.07 ± 1.218	2.41 ± 0.937	−4.239	<0.001
Unplanned reoperation			30.984	<0.001^b^
Yes	9	11		
No	33	392		
Ventilator use time (d)	2.98 ± 1.199	2.20 ± 0.837	−4.790	<0.001^a^
Urinary catheter indwelling time (d)	6.714 ± 3.884	4.645 ± 3.832	−23.326	<0.001
Central venous catheterization time (d)	6.619 ± 2.862	5.67 ± 2.775	−4.085	<0.001

a Non-parametric test.

b Fisher exact probability method.

### 3.5 Multivariate analysis

Multivariate logistic regression analysis identified diabetes, BMI, preoperative white blood cell count, intraoperative blood transfusion, duration of pericardial mediastinal drainage tube placement, total drainage volume, ventilator use duration, and central venous catheterization duration as independent risk factors for HAIs post-CABG (*p <* 0.05). The variance inflation factors were all less than 10, indicating no multicollinearity among the included independent variables. The detailed results are as shown in Table 5.

**Table 5:** Analysis of independent risk factors of HAIs post-CABG.

Influencing factor	*β* value	Standard error	Wald value	*p* value	OR value	95%CI
Body mass index	−2.238	1.006	4.955	0.026	0.107	[0.015,0.765]
Diabetes	1.467	0.718	4.174	0.041	4.338	[1.061,17.726]
Preoperative white blood cell count (×10^9^/L)	0.117	0.043	7.518	0.006	1.124	[1.034,1.222]
Preoperative albumin (g/L)	−0.146	0.073	3.981	0.046	0.864	[0.749,0.997]
Preoperative c-reactive protein (ng/L)	0.034	0.017	3.790	0.052	1.035	[1.000,1.071]
Operation duration (h)	0.213	0.197	1.171	0.279	1.237	[0.841,1.820]
Intraoperative blood transfusion volume (ml)	0.001	0.000	4.422	0.035	1.001	[1.000,1.002]
Unplanned operation	1.515	1.765	0.736	0.391	4.547	[0.143,144.709]
Addition of antibiotics during operation	3.443	2.162	2.536	0.111	31.282	[0.452,171.942]
Pericardial mediastinal drainage tube indwelling time (d)	0.864	0.201	18.483	0.001	2.372	[1.600,3.517]
Thoracic drainage tube	0.910	0.889	1.048	0.306	2.485	[0.435,14.195]
Total drainage (ml)	0.003	0.001	7.154	0.007	1.003	[1.001,1.006]
Hospital stay (d)	0.096	0.062	2.398	0.121	1.100	[0.975,1.242]
Postoperative hospital stay (d)	0.051	0.033	2.468	0.116	1.053	[0.987,1.122]
sICU time (d)	−0.283	0.353	0.639	0.424	0.754	[0.377,1.507]
Ventilator use time (d)	0.656	0.325	4.062	0.044	1.927	[1.018,3.647]
Urethral intubation time (d)	0.080	0.051	2.479	0.115	1.084	[0.981,1.197]
Central venous catheterization time (d)	0.203	0.103	3.910	0.048	1.225	[1.002,1.498]

### 3.6 Model goodness-of-fit test

A predictive model for HAIs post-CABG was developed using multivariate logistic regression, represented by the equation:

Ln (P/1-P) = −2.230 + 1.467 * diabetes + 0.117 * preoperative white blood cell count −0.146 * preoperative albumin + 0.001 * intraoperative blood transfusion + 0.864 * drainage tube indwelling + 0.003 * drainage volume + 0.656 * ventilator use time + 0.103 * central venous catheterization time.

**Figure 1:**
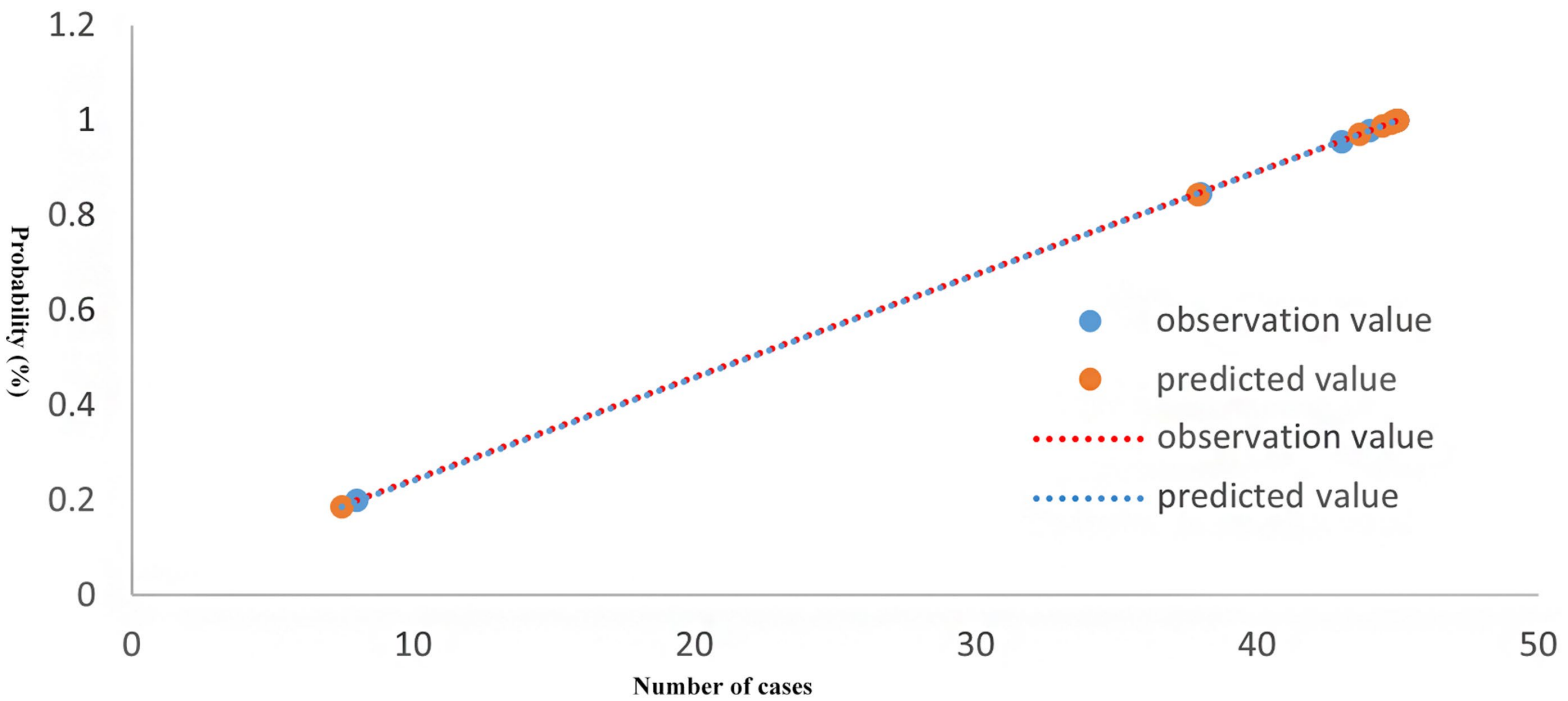
Test of goodness-of-fit of prediction model.

The Hosmer-Lemeshow test results indicated that the difference between predicted and actual values was not statistically significant (*χ^2^* = 3.032, *p* = 0.932), demonstrating a good model fit, as illustrated in Figure 1.

### 3.7 ROC analysis for predictive value of model

**Figure 2:**
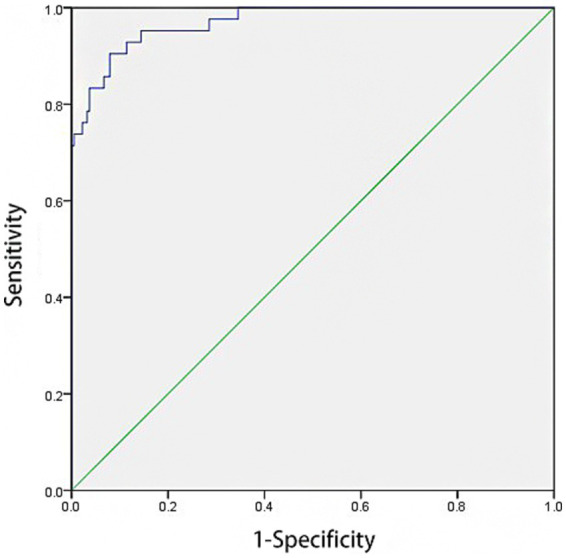
ROC curve of prediction model of logistic regression.

ROC curve analysis revealed that the model had an AUC of 0.970 [95% CI: 0.948–0.993] for predicting HAIs post-CABG. The model demonstrated a sensitivity of 90.5% and a specificity of 92.1%. The cut-off value was determined to be 0.825, with *p*-value < 0.001, as illustrated in Figure 2.

## 4 Discussion

CABG is one of the primary treatment strategies for coronary artery diseases both in China and internationally (13). As the number of surgical interventions for coronary artery diseases continues to increase annually, the incidence of HAIs following surgery is also rising (14–16). However, HAIs are largely preventable. In recent years, predictive modeling has seen widespread application in clinical practice (17). Early identification of these risk factors allows for timely clinical interventions, thereby reducing the incidence of postoperative HAIs.

Previous studies have shown that the incidence of HAIs ranges from 3 to 5% in developed countries, while in developing countries it may reach 10 to 20%. In this study, the HAI rate among CABG patients was 9.44%, lower than the 28.10% reported by Zhao et al. (18) but higher than the 2.74% reported by Qi et al. (19). Variations may be attributed to differences in study periods, patient populations, geographic locations, and sample sizes. The most common infection site was the respiratory tract, consistent with findings from other domestic studies (18, 19). International research also indicates that respiratory and urinary tract infections are the most frequent HAIs following CABG (20), further confirming the lower respiratory tract as the predominant infection site.

Intraoperative and early postoperative tracheal intubation disrupts the airway’s natural defenses and hinders secretion clearance, increasing the risk of respiratory infections. These findings suggest that targeted respiratory infection prevention strategies should be implemented post-CABG. The primary pathogens identified were Gram-negative bacteria, aligning with domestic and international trends (21, 22). While Gram-negative organisms should be prioritized in empirical therapy, variation in specific pathogens across regions and time points underscores the need for antimicrobial stewardship based on local susceptibility profiles. These results provide evidence for the rational use of antibiotics in clinical settings.

By using logistic regression, we constructed a predictive model for HAIs in CABG patients. The model identified several independent risk factors: diabetes, body mass index, preoperative white blood cell count, intraoperative blood transfusion volume, duration of pericardial and mediastinal drainage tube placement, total drainage volume, duration of mechanical ventilation, and central venous catheterization time. Previous studies suggest that comorbidities and immunosuppression increase HAI risk (23). Invasive procedures such as intubation, catheterization, and drainage compromise the body’s natural defenses, and prolonged indwelling time or inadequate aseptic technique further elevate infection risk (24, 25). Moreover, intraoperative blood transfusions may suppress immune function (26) and introduce pathogens, thereby increasing HAI risk. This finding is consistent with Kato et al. (27). Interestingly, urinary catheterization was not identified as an independent risk factor in this study, possibly due to the limited sample size, suggesting a need for further investigation with larger cohorts.

Prevention remains the cornerstone of HAI management following CABG. Accurate risk stratification and early intervention are crucial to reducing infection rates (28). Research indicates that predictive models based on statistical analysis can accurately forecast infection events and inform targeted interventions (29). In our study, the predictive model demonstrated strong diagnostic performance, with an AUC of 0.970. The Hosmer-Lemeshow test showed good model calibration, indicating strong internal consistency and potential clinical applicability. To translate this model into practice, HAI surveillance teams can integrate it into routine monitoring of CABG patients to identify high-risk individuals earlier and implement timely infection control measures. Clinical implications include: (1) System integration: Linking the predictive model with the hospital’s electronic health system for real-time risk alerts; (2) Guideline development: Establishing stratified prevention and monitoring protocols based on patient risk levels; (3) Multidisciplinary support: Forming cross-departmental teams to resolve implementation issues and ensure smooth integration into clinical workflows.

This study has some limitations. First, it is a retrospective single-center study from a tertiary hospital, limiting generalizability and introducing potential selection bias. Second, the model has not been externally validated in other clinical settings, and its applicability requires further confirmation. Future research involving multicenter, large-sample prospective studies will aim to include cross-validation or bootstrap methods to validate the model for broader clinical use.

In conclusion, independent risk factors for HAIs after CABG included diabetes, high BMI, elevated preoperative white blood cells, greater blood transfusion volume, prolonged drainage and catheterization times, and increased drainage volume. The risk prediction model showed high accuracy and may aid in guiding infection prevention and management.

## Data Availability

The original contributions presented in the study are included in the article/supplementary material, further inquiries can be directed to the corresponding author.
